# Developing a Smartphone-Based Adjunct Intervention to Reduce Cannabis Use Among Juvenile Justice-Involved Adolescents: Protocol for a Multiphase Study

**DOI:** 10.2196/35402

**Published:** 2022-03-11

**Authors:** Sarah A Helseth, John Guigayoma, Dayna Price, Anthony Spirito, Melissa A Clark, Nancy P Barnett, Sara J Becker

**Affiliations:** 1 Department of Behavioral and Social Sciences Brown University School of Public Health Providence, RI United States; 2 Department of Psychiatry and Human Behavior Alpert Medical School of Brown University Providence, RI United States; 3 Department of Health Services Policy & Practice Brown University School of Public Health Providence, RI United States; 4 Department of Obstetrics & Gynecology Alpert Medical School of Brown University Providence, RI United States

**Keywords:** mobile intervention, juvenile justice, justice, court, adolescent, teenager, substance use, cannabis, youth, adolescence, protocol, mHealth, mobile health, substance use, user design, behavioral app, health app, development, pilot, prototype, feasibility, acceptability, smartphone app, marijuana, mobile phone

## Abstract

**Background:**

Adolescents involved in the juvenile justice system who use cannabis are at an increased risk of future substance use disorders and rearrest. Many court-involved, nonincarcerated (CINI) youth are referred for services in the community and often encounter multiple barriers to care, highlighting the need for minimally burdensome services that can be delivered in justice settings. Digital health interventions are accessible, easy to implement, and can provide ongoing support but have not been developed to address the unique needs of CINI youth who use cannabis.

**Objective:**

This multiphase study will aim to develop, implement, and pilot test a novel smartphone app, Teen Empowerment through Computerized Health (TECH), to reduce cannabis and other substance use among CINI youth. TECH is conceptualized as a digital adjunct to a brief computerized intervention delivered by our family court partner.

**Methods:**

Following the principles of user-centered design, phase I interviews with CINI youth aged 14-18 years (n=14-18), their caregivers (n=6-8), and behavioral health app developers (n=6-8) will guide the TECH design decisions. Next, in phase II, CINI youth (n=10) will beta test the TECH app prototype for 1 month; their feedback regarding feasibility and acceptability will directly inform the app refinement process. Finally, in phase III, CINI youth (n=60) will participate in a pilot randomized controlled trial for 6 months, comparing the preliminary effectiveness of the adjunctive TECH app on cannabis use outcomes.

**Results:**

Phase I data collection began in September 2020 and was completed in December 2021; 14 CINI youth, 8 caregivers, and 11 behavioral health app developers participated in the study. Phases II and III will occur in 2022 and 2023 and 2023 and 2025, respectively.

**Conclusions:**

This body of work will provide insight into the feasibility and acceptability of a smartphone-based adjunctive intervention designed for CINI youth. Phase III results will offer a preliminary indication of the effectiveness of the TECH app in reducing cannabis use among CINI youth.

**International Registered Report Identifier (IRRID):**

DERR1-10.2196/35402

## Introduction

### Background

Cannabis use is common among court-involved, nonincarcerated (CINI) youth, who comprise 74% of justice-involved youth [1]—of those who test positive for a substance at the time of their arrest, 92% test positive for cannabis [2]. Early-onset cannabis use has been linked to many negative long-term outcomes, including violent behavior [3], criminal justice involvement [4,5], and substance use (SU) disorders [6]. In addition, cannabis use is a risk factor for subsequent rearrest and detention among CINI youth, perpetuating their involvement in the juvenile justice system [7]. The synergistic, maladaptive relationship between early cannabis use and juvenile justice involvement [8] highlights the need for early interventions targeting cannabis use in CINI youth [9]. Unlike detained youth who can be funneled directly into treatment on-site [10], CINI youth are typically referred to community providers, shifting treatment-seeking responsibilities to CINI youth and their families. Families involved with the legal system often face multiple competing demands that can make it difficult to engage in treatment [11], even when court-mandated, placing CINI youth at risk for escalating legal consequences and SU problems. Recent research indicates that only 28% of detained youth [12] and <30% of CINI youth [13] in need of SU services ultimately receive any treatment. Given that many court agencies were not designed to deliver SU treatments, on-site services need to minimize resource demands, maximize accessibility, and above all, be effective.

Although research on effective interventions to reduce cannabis use among CINI youth is in its infancy, theory and evidence from the broader adolescent SU literature may offer valuable insight. To date, theories of adolescent SU [14] have centered on several key intrapersonal and interpersonal mechanisms of SU behavior. Intrapersonal mechanisms, including one’s attitudes, self-efficacy, beliefs, and SU expectancies, are thought to be informed via didactic (eg, SU education [15]) and social (witnessing parent [16-18] or peer [19] SU) learning and later refined by direct experiences. Theorized interpersonal mechanisms of SU behavior encompass both direct influence from others, such as social reinforcement or modeling [20,21], and indirect influences, including perceived norms of peers’ SU [14,22].

Recent systematic and meta-analytic reviews of SU treatments for youth [23-25] indicate that cognitive-behavioral, family-based, and motivation-enhancing approaches are most effective at decreasing SU behaviors and problems in adolescents. Established intervention approaches primarily target intrapersonal mechanisms of SU. Cognitive-behavioral and family-based approaches can take 12 to 16 sessions [26], whereas motivation-enhancing models require fewer sessions and organizational resources, suggesting they could potentially be more cost-effective [27-29] and disseminable [30] in justice settings. Although brief motivation-enhancing approaches have shown promise among justice-involved youth [31,32], such interventions could be strengthened by incorporating components that also target interpersonal mechanisms of SU. For example, a recent randomized controlled trial (RCT) of a motivation-enhancing intervention incorporating accurate peer cannabis use norms found that participants reported more accurate peer norms after treatment [33]. In turn, changes in peer norms and one’s own approval of cannabis use were linked to posttreatment improvements in cannabis use and related outcomes. Finding new ways to foster peer engagement and build supportive communities in adolescent treatment contexts akin to SU recovery (eg, 12-step programs, peer recovery specialists, and online support groups) could prove to be a powerful means of promoting SU behavior change among youth.

Digital health interventions that combine motivation-enhancement and accurate peer norms may be especially well-suited to treat CINI youth in justice settings because they are accessible, customizable, portable, and show evidence of reducing cannabis use [34-39]. Among adolescents who receive brief computerized SU treatments [40], effects appear to fade over time [41], suggesting that adjunct treatments may be needed to help sustain gains. Smartphone apps are an especially appealing means of providing youth ongoing, on-demand care as an adjunct to concurrent or recently completed services (eg, continuing care and recovery support). More than 91% of US youth have their own smartphones [42], which they use for everything from schoolwork to seeking support from peers during challenging times [43]. Overwhelming evidence shows that teens find mobile apps to be a confidential and acceptable intervention platform, one with which they engage much more frequently than adults [44,45].

Most publicly available smartphone apps for SU do not incorporate evidence-based practices or resources related to SU treatment [46,47], but the availability of empirically-supported apps is increasing. A 2021 systematic review of smartphone apps for SU [48] identified 2 adjunctive smartphone apps specifically for youth with cannabis use. Although studies of both apps established the feasibility and potential efficacy of adjunctive smartphone apps for cannabis use [44,49-52], these apps were built for older, heavy cannabis users who were also receiving in-person therapy from skilled clinicians, and neither was designed for youth in low-resource settings like the juvenile justice system. Critically, no existing apps built to help youth reduce cannabis or other SU have any peer networking features. In other words, no currently available app designed for youth who use substances leverages interpersonal mechanisms of change. This represents a significant limitation to existing apps as well as an opportunity to advance the field. Therefore, new research is needed to establish the feasibility, acceptability, and effectiveness of an adjunct smartphone app targeting intrapersonal and interpersonal mechanisms to reduce cannabis use among high-risk teens in low-resource settings.

### Study Aims and Hypotheses

This protocol aims to develop, feasibility test, and evaluate the preliminary effectiveness of Teen Empowerment through Computerized Health (TECH) app as an adjunct to standard family court services (ie, treatment-as-usual [TAU]) to reduce cannabis and other SU among CINI youth. We conceptualize TECH as a peer-facilitated adjunct to brief, motivation-enhancing treatments for cannabis use—a virtual community where CINI youth can give and receive ongoing support around SU-related behavior change. Study activities will address three scientific aims and two hypotheses:

- Aim 1: develop a user-driven smartphone app, TECH, to reduce cannabis use among CINI youth
- Aim 2: examine the feasibility and acceptability of TECH
  *Hypothesis 1*: TECH will be feasible for and acceptable to CINI youth
- Aim 3: test the preliminary effectiveness of TECH as an adjunct to TAU (ie, TAU-only vs TAU+TECH) on cannabis and other SU (primary outcome)
  *Hypothesis 2*: CINI youth who receive TAU+TECH will demonstrate greater reductions in cannabis and other SU-related outcomes relative to TAU-only CINI youth. We will also explore possible intervention effects on a secondary outcome (ie, delinquent behavior) and multiple putative mediators (ie, intrapersonal and interpersonal mechanisms of change)
  *Exploratory Hypothesis 1*: youth in the TAU+TECH group will show greater change on the secondary outcome and putative mediators

## Methods

### Study Overview

The study aims will be achieved across 3 distinct study phases. Phase I will consist of qualitative interviews with CINI youth (n=14-18), their caregivers (n=6-8), and behavioral health app developers (n=6-8) to determine how, why, and when CINI youth would most prefer to engage with a smartphone app to reduce cannabis use. Qualitative results will guide decision-making on the key features and overall design of the TECH app, in partnership with our software developer. Phase II will beta test the TECH app with CINI youth (n=10) to guide decision-making to refine the app. Phase III will encompass a pilot RCT with CINI youth (n=60) to compare the treatment effects of TAU-only and TAU+TECH.

### Theoretical Frameworks

The study will be guided by 2 frameworks. First, the research approach will follow principles of user-centered design [53] for accelerating digital health research [54]. In this context, the user-centered design approach will engage key stakeholders in all aspects of TECH development and refinement, to ensure the resulting app is tailored to the specific needs and preferences of CINI youth who use cannabis. Second, the Behavior Intervention Technology (BIT) model will guide the research team’s efforts to build the TECH app prototype [55]. The BIT model helps researchers clarify why a digital health product is needed (eg, clinical and usage aims for the TECH app), how it is expected to change users’ behavior (eg, interpersonal and intrapersonal mechanisms), and what technical components will achieve the desired effects (eg, elements, characteristics, and workflow of the TECH app features).

### Study Setting

This research will be done in partnership with a state-wide family court in the Northeastern United States. The partner family court has jurisdiction over all delinquent, wayward, dependent, psychiatrically disordered, and diverted youth aged <18 years.

### Standard of Care Services

All CINI youth who participate in any phase of this study will receive standard services from the family court, including juvenile intake and any indicated referrals. In the juvenile intake, court staff workers screen youth for mental health and SU problems. Intake workers then make referrals; options for continued system involvement include formal delinquency charges, diversion programs (eg, mandated community service, mental health services, or SU services), ongoing supervision, or probation.

In 2017, the family court began recommending the eCHECKUP-TO-GO (ECTG) program for cannabis [56] as their standard of care for CINI youth who present with a history of cannabis use. ECTG is a computerized single-session, motivation-enhancing intervention that generates personalized behavior change goals related to cannabis and SU across personal, academic, and social domains and has shown positive outcomes among cannabis-using [37,57] and abstinent [58] college students. All CINI youth who participate in any phase of this study will receive the computerized ECTG program for cannabis use.

### Inclusion Criteria

Three types of participants will be recruited: CINI youth, their caregivers, and behavioral app developers. Notably, caregivers of CINI youth and CINI youth will participate independently in the study (ie, we are not enrolling caregiver+CINI youth dyads). CINI youth will be recruited for all study phases and must be (1) aged 14-18 years, (2) able to speak and read English, (3) have access to a smartphone, (4) report past-year cannabis use and screen positive on either the CRAFFT (car, relax, alone, forget, friends, trouble) [59] or the Massachusetts Youth Screening Instrument-Version 2 (MAYSI-2 [60])—the family court’s preferred evidence-based SU screening measures, and (5) have a caregiver willing and able to provide consent for their participation. In phases II and III, eligible CINI youth must also be (6) willing to adhere to the TECH app’s user safety agreement during the consent process. Importantly, caregivers providing consent for CINI youth to participate must be able to complete the consent process in English or Spanish. Caregivers and behavioral health app developers will only participate in phase I interviews. Caregiver participants must (1) speak in English and (2) be a legal guardian and primary caregiver of an eligible CINI youth. Behavioral health app developers must (1) speak and read in English and (2) be lead developers of a research-based smartphone app targeting behavioral health outcomes. Exclusion criteria are limited to enhance generalizability and only include conditions that preclude CINI youth from actively participating in an interview or using the proposed app (eg, psychosis, cognitive impairment, no smartphone access, or non-English speaking).

### Recruitment

CINI youth (phase I, n=14-18; phase II, n=10; and phase III, n=60; in total, n=84-88) will be recruited through the family court’s juvenile intake department. Court intake staff will receive a brief training on the study objectives, eligibility criteria, and referral procedures. Upon screening a study-eligible adolescent, intake staff will describe whichever phase of the study is actively recruiting participants; CINI youth aged >18 years and caregivers of interested minors will sign a consent-to-contact form allowing contact with the investigators. The phase I consent-to-contact form allows families to indicate whether the CINI youth, the caregiver, or both would be interested in interviewing. When recruiting CINI youth who are minors, the research team will initiate contact with their caregivers.

Caregivers of CINI youth (phase I only; n=6-8) will be recruited through the family court’s juvenile intake department via the same methods used to recruit CINI youth. Following receipt of the consent-to-contact form, the principal investigator will contact eligible caregivers to describe the study interviews.

Behavioral health app developers (phase I only; n=6-8) will be identified via searches of the literature and repositories of federally funded grants to ensure they were a lead developer of a research-based smartphone app targeting behavioral health outcomes. Given TECH’s focus, preference will be given to developers of youth- or SU-focused apps. The principal investigator will contact eligible individuals to describe the study and invite them to reply if interested.

### Compensation

All participants will receive financial compensation for their participation in the study. Phase I participants will receive a US $50 gift card upon completion of the study interview. Phase II and III CINI youth will receive up to US $90 and US $200 in gift cards, respectively.

### Retention

To promote retention of phase II and III CINI youth at follow-up, we will collect multiple sources of contact information at baseline, including social media handles [61]. Participants will also receive gift cards escalating in value for each completed assessment (ie, US $40 at baseline, US $50 at 1 and 3 months, and US $60 at 6 months).

### Informed Consent

#### Overview

All participants will be fully informed of the purposes and procedures of the study, both verbally and through a written description in assent and consent forms. Individuals aged >18 years (ie, caregivers, behavioral health app developers, and CINI youth who are legally adults) will provide informed consent before study enrollment; for minor CINI youth, we will obtain consent from their legal guardian and assent from the youth. The informed consent and assent processes will be conducted in English by a research team member; a bilingual, trained research assistant will complete the process with any Spanish-speaking caregivers providing consent for minor CINI youth. This process will occur either in-person, by phone, or via videoconference, depending on the potential participant’s preference.

Potential participants and caregivers providing consent for CINI youth will be reminded that their participation is strictly voluntary, they can refuse to answer any questions, and they may withdraw from the study at any time. Potential adolescent and caregiver participants will also be assured that they may decide to participate, not participate, or withdraw from the study without fear of penalty from the family court. To ensure adequate comprehension, a research team member will ask potential participants to describe the purpose of the study and the basic study procedures. If the potential participant does not appear to understand the study, the research team member will read aloud key portions of the consent or assent form and provide brief verbal summaries of each section of the consent or assent form. Once adequate understanding has been demonstrated, potential participants will be asked to electronically sign their consent or assent form via a Qualtrics survey. Participants will be sent a copy of their completed form, for their records.

#### User Safety Agreement

As part of the consent/assent process, phase II and III CINI youth will also be required to agree to adhere to a user safety agreement describing appropriate use of the TECH app. Digital and social media products often require users to agree to adhere to specific terms regarding their use; researchers have begun to include such agreements in their technology-based studies [62]. The TECH app user safety agreement will specify guidelines for appropriate peer interactions in the digital research context (eg, maintaining privacy and anonymity, respect for others, relevant content, the role of the moderator, and protecting the safety of all users) and outline possible consequences for violating the terms of use (eg, moderators will delete posts and remove the participants’ access to interactive app features).

### Sample Size Feasibility

We aim to recruit 84 to 88 CINI youths throughout the 5-year, multiphase protocol. Approximately 1500 youths are referred to the family court annually. In a recent study of a sample of youth screened by the family court, 173 (50%) of 348 youths screened positive for problematic cannabis use [63], indicating that the recruitment pool is sufficiently robust to support the proposed recruitment targets.

### Phase I: Formative Research and TECH Prototype Development

#### Overview

Semistructured interviews will be conducted with CINI youth (n=14-18), their caregivers (n=6-8), and behavioral health app developers (n=6-8). The primary goal is to gather data from CINI youth, to guide decision-making and prioritization of the TECH app features. Broadly, the interviewers will uncover potential barriers to app development or implementation.

#### Interview Guides

Separate interview guides will be used for each type of participant, but all will explore the following BIT [55] model dimensions: (1) clinical aims, to learn what types of intervention goals would be most relevant and appealing to CINI youth (eg, avoid arrest and decrease cannabis use); (2) usage aims, to understand how CINI youth use behavioral health apps; (3) behavior change strategies, to identify which theory-driven approaches to behavior change appeal most to CINI youth (eg, goal-setting, monitoring, education, and peer support or advice); (4) elements, to identify those app features most relevant and appealing to CINI youth to address their cannabis use (eg, notifications, text messages, and newsfeed); (5) characteristics, to establish CINI youths’ preferences around personalization, complexity, types of media employed, and general esthetics (eg, settings, design, and individualization); and (6) workflow, to understand CINI youths’ preferred conditions for engaging with an app (eg, time- vs goal-based) to define a user-driven workflow.

Interviews with behavioral health app developers will focus on their experiences developing, implementing, and refining smartphone apps for behavioral health. Caregiver interviews will capture thoughts about their teen’s potential use of a smartphone app for cannabis use, potential barriers to their teen’s ongoing use of the app, and the perceived feasibility and acceptability of digital health interventions for high-risk teens.

CINI youth participants will begin their interviews upon completing the computerized ECTG program for cannabis [56] (ie, TAU). Youth will describe their access to and use of smartphones, how they engage with peers digitally, and share their perceptions of and prior experience with behavior change apps, to capture the feasibility and acceptability of digital behavioral health interventions for CINI youth. Interviews will examine youths’ concerns about using smartphone-based interventions in juvenile justice contexts, including security and privacy, court staff access to the app, and perceived risks of connecting digitally with other CINI youth. The bulk of the interview will be dedicated to BIT [55] domains to inform how the proposed TECH app could best support CINI youth enacting ECTG-recommended changes in cannabis use and other behaviors. Youth will rate each app feature on a 10-point Likert scale of importance and name their top 3 preferred features, to ensure the TECH app features align with CINI youths’ preferences.

#### Interview Procedures

All interviews will be conducted and audio recorded by the principal investigator (SH). Behavioral health app developer and caregiver interviews will take 30 to 60 minutes, whereas CINI youth interviews will take 60 to 90 minutes. Participants will complete any quantitative measures before their interviews.

#### Interview Quantitative Measures

All participants will be asked to provide basic demographic information (eg, age, biological sex, and education level).

CINI youth will be asked to describe their recent history of behavioral health services via the Child and Adolescent Services Assessment [64,65]. The Marijuana Use Questionnaire [66,67] will capture youths’ preferred type (eg, plant or concentrate) and mode (eg, vape, smoke, and edible) of cannabis use. Youth will also report on their perceptions of normative cannabis use among their peers. Following standard practices [33,68], three types of norms will be captured for two reference groups (ie, your close friends and other teens your age): (1) descriptive norms ask participants to estimate, within each reference group, the percentage of youth who have ever used cannabis and who use cannabis regularly, along with their past-month and past-year frequency of use, on a 9-point Likert scale (0=*never* to 8=*every day*); (2) injunctive norms, which assess each reference group’s approval of youth who have never used cannabis, used once or twice, use occasionally, and use regularly, on a 7-point Likert scale (1=*strongly disapprove* to 7=*strongly approve*); and (3) subjective norms, which measure each reference group’s perceived approval of the participant’s past 30-day cannabis use on a 7-point Likert scale (1=*strongly disapprove* to 7=*strongly approve*).

#### Interview Analysis

All phase I quantitative measures will be analyzed using percentages or means and SDs. These data will offer descriptive insights into each group of interviewees.

Interviews will be transcribed and checked by 2 research team members to identify any discrepancies before the analysis. Data from each type of participant will be analyzed separately using the following directed content analysis [69] approach. The analysis will begin with an a priori coding framework derived from BIT model constructs but will allow for the emergence of unanticipated codes. First, 2 team members will independently code the same transcript; the research team will convene to discuss identified codes to refine BIT codes and reach consensus regarding emergent codes. This process will be completed for each type of participant, resulting in a series of 3 codebooks. All transcripts will then be independently coded by 2 team members. The team will reconvene to discuss divergent codes with the goal of achieving 100% consensus; codes that remain discrepant following this discussion will be decided by a third, independent coder. Coding will occur on a rolling basis, to allow for periodic assessment of data saturation [70,71].

#### Building the TECH App

##### Anticipated Components

Although our final decision-making will be driven by phase I formative work, we anticipate including several theory-driven components in the TECH app. Specifically, in-app goal-setting and behavior tracking (eg, promoting self-efficacy) as well as delivery of accurate SU information (eg, helping to change attitudes and beliefs about SU) will leverage intrapersonal mechanisms of change. Interpersonal mechanisms of change will be targeted through a newsfeed for youth to post their progress (eg, modeling and social norms) and receive peer support (eg, social reinforcement). To protect against deviant peer influence [72], the app will be programmed to prevent negative (eg, *thumbs-down*) or covert (eg, direct messaging) features, and the moderator will monitor in-app activity daily.

##### App Development

**Figure 1:**
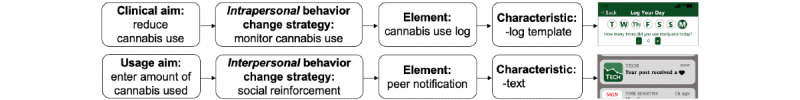
Applying the behavior information technology model [55] to the proposed Teen Empowerment through Computerized Health app.

The TECH app prototype will be built in partnership with Mooseworks Software LLC, a development company that builds apps from a software library of preprogrammed features, including, but not limited to, networking forums, community newsfeed, push notifications, tracking logs, and sharable posts. Guided by phase I formative work, the research team will engage in a series of meetings to select core elements and features of TECH in consultation with the software developer. Following the BIT model [55] (Figure 1), this process will match theory-driven intrapersonal (ie, self-efficacy, attitudes, and beliefs about SU) and interpersonal (ie, modeling, social norms, and social reinforcement) mechanisms of behavior change with CINI youth’s preferred app features. The research team will test the prototype’s functionality before launching phase II. In addition, the research team will populate the app with content solicited during phase I interviews with CINI youth to ensure the app is engaging to initial participants.

### Phase II: Beta Testing the TECH Prototype

#### Overview

In the next phase of the study, CINI youth (n=10) will beta test the TECH prototype. Beta testing enables users to identify and refine unclear components and troubleshoot software bugs. Methodologically, beta testing helps researchers reduce dissatisfaction, assess acceptability, and test and refine key study procedures (eg, app introduction, user safety agreement adherence, and metadata collection) before an RCT [73-76].

#### Beta-Testing Procedures

CINI youth recruitment, caregiver consent, and assent procedures will be identical to phase I, with one exception. Phase II will use a phased approach to recruitment, wherein the research team will wait to schedule baseline assessments until receiving consent-to-contact forms for 4 CINI youths. This will help ensure sufficient in-app peer presence for initial participants.

Enrolled CINI youth will attend a study session to complete baseline measures, the computerized ECTG program for cannabis use [56] and receive an introduction to the TECH prototype. A research team member will help participants download the app, create an anonymous account and log in, review the TECH app user safety agreement, and learn how to use each app feature. The research team member will help teenagers enter at least two cannabis-related behavior change goals derived from the ECTG program [56]. The TECH app introductions will be audio recorded to assess researcher adherence to the introduction protocols. The research team will monitor the TECH app activity daily to ensure prompt handling of any app functionality problems or participant concerns.

CINI youth will be asked to use the TECH app prototype for 1 month, completing a brief measure of acceptability each week. At the end of the beta-testing period, participants will complete a series of post–beta-testing measures of feasibility and acceptability. The TECH app metadata will be examined on an ongoing basis to monitor use patterns, note which app features are underused, and identify opportunities for targeted intervention to promote increased use. To test urine drug screen administration procedures for phase III’s pilot RCT, phase II CINI youth will be asked to complete a Clinical Laboratory Improvement Amendments–waived urine drug screen with eight panels (ie, cannabis, amphetamines, methylenedioxy-methylamphetamine, opiates, oxycodone, methamphetamines, cocaine, and benzodiazepines) during their post–beta-testing appointment. For in-person appointments, a research team member will request a urine sample and administer the dip test. Youth who complete appointments virtually will be mailed necessary screening materials before their appointment and receive step-by-step instructions to self-administer the dip test. Once the results are recorded, all the specimens and testing materials will be destroyed.

#### Beta-Testing Measures

##### Baseline

Measures will be identical to those used in phase II.

##### Feasibility

The feasibility of the TECH app prototype will be assessed using two primary indicators: (1) percentage of CINI youth willing to participate (recruited vs enrolled) with a target of 50% and (2) TECH usability, measured by automatically collected metadata. Available metadata will depend on design decisions following phase I formative work (eg, forum or newsfeed needed to make a post). Ideal app use would involve at least half of phase II participants using the app 2 or more times, spending an average of 30 seconds in the app when using it, posting at least once, and liking a peer’s content at least once.

##### Acceptability

We will measure the acceptability of the TECH app via four quantitative indicators: (1) percentage of CINI youth who withdraw from the beta-testing process, with a target of less than 20%; (2) weekly administration of the user version of the Mobile Application Rating Scale [77], an established, 20-item measure of an app’s engagement, functionality, esthetics, and information quality, with a targeted app quality mean score of 2.5, or higher (out of 5); (3) the Consumer Satisfaction Questionnaire [78], with a target of at least 70% of CINI youth indicating that they are satisfied and would recommend TECH to others; and (4) a structured, open-ended questionnaire to solicit feedback about the TECH prototype, including changes to content or features that could improve usability and engagement, administered at the end of the 1-month beta-testing period.

#### Beta-Testing Analysis

Due to the small sample size, phase II measures will only be used to offer insight into the feasibility of study procedures and guide the TECH app refinement. Data will be presented in aggregate and analyzed using means, SDs, or similar types of statistical analyses (eg, percentages).

#### Refining TECH

Similar to the development of the prototype, the TECH app refinement will follow the BIT model [55] and incorporate feedback provided by CINI youth during beta testing. The research team will work with our software developer to troubleshoot usability issues, consider modifications to address acceptability concerns, and finalize the TECH app.

### Phase III: Pilot RCT of the Final TECH App

#### Overview

In the final phase of the study, we will conduct a pilot RCT with CINI youth (n=60) to examine the feasibility, acceptability, and preliminary effectiveness of the TECH app. CINI youth recruitment, caregiver consent, assent, and baseline appointment procedures will be identical to those in phase II. Table 1 depicts the timing of phase III activities.

**Table 1:** Schedule of enrollment, interventions, and assessments for phase III’s pilot randomized controlled trial.

		Enrollment, -t_1_	Allocation	Postallocation				Closeout, t_x_^a^
				t_1_^b^	t_2_^c^	t_3_^d^		
**Enrollment**								
	Screening	✓						
	Informed consent or assent	✓						
	User Safety Agreement	✓						
	Baseline	✓						
	Randomization		✓					
**Interventions**								
	TAU^e^ (all study participants)	✓						
	TECH^f^ app (only TAU+TECH participants)			✓	✓	✓		
**Phase III measures for all participants**								
	Demographics	✓						
	Child and Adolescent Services Assessment [64,65]	✓						
	Marijuana Use Questionnaire [66,67]	✓						
	Timeline Followback Interview [79-81]	✓		✓	✓	✓		
	Urine Drug Screens	✓		✓	✓	✓		
	Selected items on Global Appraisal of Individual Needs [82]	✓		✓	✓	✓		
	Selected items on cannabis use attitudes [83]	✓		✓	✓	✓		
	Marijuana Adolescent Problem Inventory [84]	✓		✓	✓	✓		
	Marijuana Effect Expectancy Questionnaire [85]	✓		✓	✓	✓		
	Cannabis Refusal Self-Efficacy Questionnaire [86,87]	✓		✓	✓	✓		
	Adapted Readiness to Change Questionnaire [88]	✓		✓	✓	✓		
	Descriptive, Injunctive, and Subjective Peer Norms [33]	✓		✓	✓	✓		
**Phase III measures completed by TAU+TECH participants**								
	Mobile Application Rating Scale (user version) [77]			✓	✓	✓		
	Adapted Consumer Satisfaction Questionnaire [78]			✓	✓	✓		
	Open-ended TECH app feedback questionnaire			✓	✓	✓		
**Other phase III measures not completed by participants**								
	Percent of CINI^g^ youth recruited vs enrolled						✓	
	Percent of CINI youth who withdraw from the study						✓	
	TECH app usability metadata						✓	

^a^Conclusion of study.

^b^1 month after baseline.

^c^3 months after baseline.

^d^6 months after baseline.

^e^TAU: treatment-as-usual.

^f^TECH: Teen Empowerment through Computerized Health.

^g^CINI: court-involved, nonincarcerated.

#### Pilot RCT Randomization

Upon completing the computerized ECTG program for cannabis use [56] at the baseline appointment, participants will immediately be randomized to 1 of the 2 treatment conditions. Participants will be randomized using an urn randomization spreadsheet [89], balancing for biological sex and frequency of cannabis use. Youth will be assigned at a ratio of 2 TAU+TECH to 1 TAU-only to ensure sufficient peer presence on the TECH app at any given time.

#### Pilot RCT Treatment Conditions

##### TAU-Only

The computerized ECTG program for cannabis use [56] and the family court’s standard care services will serve as the TAU-only condition in the pilot RCT.

##### TAU+TECH

Participants in the TAU+TECH arm will receive all TAU components and access to the TECH app. Immediately after randomization, a research team member will introduce participants to the TECH app using the same procedures used in phase II. TAU+TECH participants will be asked to use the TECH app for 6 months.

#### Pilot RCT Measures

Baseline, preliminary effectiveness, and putative mediator outcome measures will be administered to all phase III participants at all time points. Feasibility and acceptability measures specific to the TECH app will only be administered to participants randomized to TAU+TECH at the 1-, 3-, and 6-month follow-up appointments.

##### Baseline

All phase II baseline measures and phase III measures of preliminary effectiveness, secondary effectiveness, and putative mediators will be administered at baseline.

##### Preliminary Effectiveness Outcomes

These measures will be completed by all phase III participants at all assessments and will assess the preliminary effectiveness of TAU+TECH relative to TAU-only. The primary outcome measure will be CINI youths’ self-reported cannabis and other SU, as measured by the Timeline Followback Interview [79-81]. Youth will report their total days of cannabis and other SU, abstinence, high-volume use, co-use, and cannabis grams per day in the past 30 days. Youth self-reported SU will be corroborated by urine drug screens using the same procedures outlined in phase II unless adjustments are deemed necessary.

##### Secondary Effectiveness Outcomes

We will collect data on delinquent behavior to account for its bidirectional relationship with cannabis and other SU. Furthermore, 9 items adapted from the Global Appraisal of Individual Needs-Core [82] will assess past 30-day delinquency and legal system involvement.

##### Putative Mediators

We will collect six indicators of putative intrapersonal mechanisms of change: (1) attitudes toward cannabis use will be assessed by two items [83] that capture the perceived acceptability of personal cannabis use on a Likert scale, one positively framed (“Is it ok...?”) and one negatively framed (“How wrong is...?”); (2) the Marijuana Adolescent Problem Inventory [84], which includes 23 items on perceived cannabis-related consequences, rated on a 0-4 Likert scale; (3) The 6-item Marijuana Effect Expectancy Questionnaire-Brief [85], which gauges positive and negative expectations of cannabis use [90]; (4) the Cannabis Refusal Self-Efficacy Questionnaire [86,87], which uses 14 items measuring self-confidence to resist or refuse cannabis; (5) an adapted Readiness to Change Questionnaire [88], which will assess motivation to reduce cannabis use with 12-items; and (6) descriptive, injunctive, and subjective peer norms of cannabis use [33,68] as collected at baseline in prior phases.

##### Feasibility and Acceptability

These measures will be identical to those used in phase II; however, only CINI youth randomized to the TAU+TECH condition will be asked to complete measures regarding their experiences using the TECH app.

#### Pilot RCT Statistical Analysis

We will conduct preliminary analyses on key variables to examine distributional properties, identify outliers, and transform variables as needed. The conditions (ie, TAU-only vs TAU+TECH) will be compared on baseline demographic variables; differences will be controlled in subsequent analyses. Data analysis will follow intent-to-treat principles [91] and use multiple imputation methods [92] in the event of unplanned missing data. Due to the small sample size, we will be underpowered for significance testing. Instead, our goal is to obtain data on variable distributions, reliability, and effect size estimates for a future large-scale trial. For the same reason, we will not attempt to test for mediation but instead test associations with possible mediators. Findings will inform which variables to evaluate in a future trial.

We anticipate 80% retention [93] of phase III CINI youth (n=60), leaving 48 participants with complete data. This sample will be sufficient to determine the feasibility and acceptability outcomes. Primary preliminary effectiveness outcomes include cannabis and other SU. Putative mediators include intrapersonal and interpersonal mechanisms of change; delinquent behavior is the sole secondary outcome. Brief motivation-enhancing treatments produce short-term decreases in cannabis and SU [40,41], but technological adjuncts can help maintain reductions in SU for up to 6 months [94]. Thus, we expect CINI youth to report the highest rates of cannabis and other SU at baseline, with small to moderate decreases at 1 month that maintain at 6 months.

We will conduct a series of repeated measures analyses of covariance to predict outcomes by condition, test putative mediators, and control for baseline differences. We will also analyze dosage effects on each dependent variable and calculate partial eta squared (*η^2^*) to estimate proportions of variance associated with TECH. Despite effect size stability concerns in small samples [95], detection of small to moderate treatment effects on our primary outcomes alongside strong evidence of feasibility and acceptability could indicate preliminary efficacy of the TECH app as an adjunct to TAU. If such evidence is found, effect size estimates will be used to determine the sample size for a future, fully-powered RCT (eg, for power of 0.8, a small effect size [Cohen *d*=0.3] would require n=175 per group, whereas a medium effect size [Cohen *d*=0.5] would require n=64).

### Ethical Oversight

The proposed study activities will be reviewed and approved by the institutional review board at Brown University. The research team has extensive experience conducting intervention research with CINI youth who use cannabis and addressing emergent safety concerns. Study activities will include several safeguards to ensure that the proposed research presents minimal risks of psychological discomfort, coercion, legal, and loss of privacy or confidentiality to CINI youth participants. Psychological discomfort will be minimized by (1) emphasizing that youth will not be obligated to answer distressing questions and can withdraw from the study at their discretion; (2) avoiding direct questions about parental neglect, abuse, or suicidal ideation; and (3) conducting a thorough safety assessment if youth spontaneously disclose these concerns. The risk of coercion will be minimized by (1) reminding CINI youth their participation is voluntary; (2) ensuring that they receive multiple referral options for substance misuse from their court intake worker, of which the study will be one; and (3) offering compensation rates consistent with previous studies and commensurate with the level of effort required. Legal risks will be minimized by obtaining a certificate of confidentiality from the National Institutes of Health and explaining its limitations. Potential loss of privacy or confidentiality will be minimized by (1) using electronic consent and assent forms instead of needing to store and/or transport hard copies from the family court to the university; (2) deidentifying participant data; (3) storing all electronic data, including audio recordings, on a password-protected, secure shared drive; (4) allowing only the principal investigator, coinvestigators, and research team members to access data files; and (5) destroying all audio recordings and any other identifiable information within 6 months of the study’s conclusion.

### Clinical Trial Registration

Our pilot RCT will be submitted for registration to the ClinicalTrials.gov website within 21 days of enrolling the first phase III participant (anticipated in summer 2023).

## Results

The study received institutional review board approval in August 2019 and was funded in March 2020. Participant recruitment began in September 2020, and phase I was completed in December 2021. A total of 11 behavioral health app developers, 8 caregivers, and 14 CINI youth completed the phase I semistructured interviews. We interviewed 3 more behavioral health app developers than initially planned to achieve data saturation. Phase I data analysis and the TECH app development were actively underway at the time of this submission. Data collection is scheduled to begin in spring 2022 and summer 2023 for phases II and III, respectively. Phase III analyses are expected to be completed in spring 2025.

## Discussion

### Overview

This multiphase research aims to develop and pilot test an adjunctive smartphone app to reduce cannabis and other SU among CINI youth. Given the high prevalence of cannabis use in this population [2] and well-documented associations with health and legal consequences later in life [3-6], preventive and early interventions are vital to disrupt the cycle of SU and criminal justice involvement among CINI youth. We believe that the proposed TECH app will be the first designed specifically for CINI youth, who represent nearly 75% of all youth involved in the juvenile justice system [1]. Although a handful of smartphone apps have been developed to support adolescents in treatment for cannabis use [44,49-52], TECH will be one of the first apps designed for youth who are not currently receiving traditional SU treatment. Treatment rates are generally low across the juvenile justice system: only 28% of detained youth [12] and <30% of CINI youth [13] in need of SU services receive any treatment.

Brief, computerized interventions such as the ECTG program [56] can deliver evidence-based content with high fidelity, making them ideal for self-administration or delivery by a workforce without behavioral health training. However, similar to most brief approaches, gains achieved through brief computerized interventions can fade over time, including in samples of adolescents who use substances [40,41]. Digital adjuncts, like the TECH app, could provide a light touch of ongoing support to help bolster treatment gains. Although this app will be tailored to address the unique needs and preferences of CINI youth who use substances, evidence supporting the feasibility and acceptability of our multimodal digital approach could have broader implications. Fully digital approaches represent a promising, if untested, solution for high-need, low-resource settings, especially those with the existing infrastructure to identify individuals at risk but lacking the workforce, capacity, or overarching mission to deliver behavioral health services, such as juvenile-court settings. Finally, despite overwhelming evidence indicating that peers play a key role in the development of adolescent SU, this study marks one of the first efforts to leverage interpersonal mechanisms of change through a peer networking forum. The proposed platform will connect youth in a monitored, virtual community that limits risky peer behavior so that they can engage and support each other’s cannabis-related behavior changes.

### Limitations

We note the following limitations to the proposed work. First, although caregiver- and family-based interventions for adolescent SU are well-established [24], this study will intervene solely on CINI youth. If TECH shows promise as an adjunct to brief computerized treatments (ie, the ECTG program for cannabis use [56]), an appropriate next step could be to deliver TECH alongside family-focused digital approaches [96]. Second, all CINI youth will receive TAU, which includes the computerized ECTG program for cannabis use [56] that has been shown to reduce cannabis use among young adults [37,57,58]. This ensures that CINI youth in-need would not be denied standard services. Although this may impact our ability to detect preliminary intervention effects specific to the TECH app, it is also the only way to assess whether the adjunctive TECH app can help sustain cannabis and other SU behavior change among CINI youth. Third, CINI youth and caregivers may be hesitant to disclose youth SU and delinquent behavior in qualitative interviews and surveys. To address this issue, our consent procedures will explain our protocols to maintain confidentiality and how study data will not lead to reprisals from family courts. Finally, although sufficient to achieve the proposed aims, the small sample in the pilot RCT may provide limited opportunities for in-app peer engagement, which may hinder interpersonal mechanisms of behavior change. We will take several steps to maximize peer presence on the TECH app, including randomizing youth 2:1 to receive the TECH app, phasing recruitment so that multiple participants receive the app concurrently, and populating the app with content collected from previous participants.

## Appendix

Multimedia Appendix 1 Peer review reports.

## Abbreviations

BIT: Behavior Intervention Technology
CINI: court-involved, nonincarcerated
ECTG: eCHECKUP-TO-GO
RCT: randomized controlled trial
SU: substance use
TAU: treatment-as-usual
TECH: Teen Empowerment through Computerized Health
