# Supplementary material for: Developing a Smartphone-Based Adjunct Intervention to Reduce Cannabis Use Among Juvenile Justice-Involved Adolescents: Protocol for a Multiphase Study
Source: JMIR Res Protoc. 2022 Mar 11;11(3):e35402. doi: 10.2196/35402 (PMC8957005; doi:10.2196/35402)
Supplement: Multimedia Appendix 1 [file resprot_v11i3e35402_app1.pdf]

SUMMARY STATEMENT
(Privileged Communication)

PROGRAM CONTACT:
Guifang Lao
301-443-1061
laog@mail.nih.gov

Release Date: 06/23/2019
Revised Date:

Application Number: 1 K23 DA048062-01A1

Principal Investigator

HELSETH, SARAH ASHLEY

Applicant Organization: BROWN UNIVERSITY

Review Group: IPTA
Interventions to Prevent and Treat Addictions Study Section

Meeting Date: 06/06/2019
Council: OCT 2019
Requested Start: 09/01/2019
RFA/PA: PA19-118
PCC: CC/GLA

Project Title: Development and Preliminary Testing of an Adjunct Smartphone App to Reduce Marijuana Use in Court-Involved, Non-Incarcerated Adolescents
SRG Action: Impact Score:15 Percentile:2 +
Next Steps: Visit https://grants.nih.gov/grants/next_steps.htm
Human Subjects: 30-Human subjects involved - Certified, no SRG concerns
Animal Subjects: 10-No live vertebrate animals involved for competing appl.
Gender: 1A-Both genders, scientifically acceptable
Minority: 1A-Minorities and non-minorities, scientifically acceptable
Age: 6A-Children and Adults, scientifically acceptable

Project Year	Direct Costs Requested	Estimated Total Cost
1	200,176	216,104
2	204,682	220,969
3	209,322	225,978
4	214,102	231,138
5	219,025	236,453
TOTAL	1,047,307	1,130,642

ADMINISTRATIVE BUDGET NOTE: The budget shown is the requested budget and has not been adjusted to reflect any recommendations made by reviewers. If an award is planned, the costs will be calculated by Institute grants management staff based on the recommendations outlined below in the COMMITTEE BUDGET RECOMMENDATIONS section.

1K23DA048062-01A1 Helseth, Sarah

RESUME AND SUMMARY OF DISCUSSION: This career development application requests support for the candidate to obtain training and mentorship in designing and testing substance use interventions in real world settings for youth at risk for substance use and other problems, and to develop and pilot test an adjunct mHealth app designed to promote marijuana use reduction among court-involved, non-incarcerated youth. The candidate is outstanding with a strong publication record and solid institutional commitment, the training plan is comprehensive, the mentoring team is accomplished and well-suited to the proposed work, and the focus on a technology-based intervention in this vulnerable population is highly significant and innovative. The candidate was highly responsive to the prior review, improving the application by publishing additional data in relevant areas, and strengthening the scientific rigor of the research plan by lengthening the assessment period, changing the recruitment plan to ensure a sufficient number of youth, and assessing motivation for decreasing marijuana use. Following the discussion, reviewers agreed that the strengths of this application from an outstanding candidate outweigh any minor weaknesses, and there is a high likelihood that the candidate's proposed career development and research plans will lead to a strong independent research career.

DESCRIPTION (provided by applicant): Through the research and training described in this K23 proposal, the PI (Dr. Helseth) will acquire the necessary skills to become an independent clinical researcher focused on designing and evaluating early, substance use (SU) interventions for youth on risky developmental trajectories. Marijuana (MJ) use accounts for 92% of positive drug screens among teens in the juvenile justice system (JJ) and has been linked to future arrests and SU disorders. Due to JJ capacity constraints, court-involved, non-incarcerated (CINI) youth are often referred out for treatment and encounter multiple barriers to community care. Technology-assisted interventions would enable the JJ system to treat CINI youth in-house, without overextending its workforce or resources. Brief, computerized motivational interventions are inexpensive and easy to deliver, and have been shown to reduce teens' MJ and SU. However, effects typically fade after a few months, indicating the need for an adjunct intervention to sustain behavior change. Smartphone apps are an ideal treatment platform for teens: 95% of teens own one, which they use nearly 3 hours each day. Yet the potential of smartphone apps as a means of reducing MJ use in high-risk youth has not been realized. With this 5-year K23, the PI aims to establish the needs and preferences of CINI youth ages 14-17 for an adjunctive smartphone app; examine the app's feasibility and acceptability; and establish its preliminary efficacy. The Teen Empowerment through Computerized Health (TECH) app, which targets putative intrapersonal and interpersonal mechanisms to promote MJ-related behavior change, will serve as an adjunct to treatment-as-usual (TAU) in the Rhode Island Family Court. Following the Behavior Intervention Theory (BIT) model, qualitative interviews with key stakeholders (n=30) will inform how clinical goals (knowledge, skills, motivation) will map on to TECH usage goals (goal-setting, peer networking), features (expert-moderated forum, notifications) and workflow. Next, 10 CINI youth will beta test the TECH prototype for 1 month, to guide its refinement. Finally, a pilot randomized control trial with 60 CINI youth will test the app for 6 months as an adjunct to TAU (TAU+TECH vs. TAU-only) on MJ and other SU-related outcomes, putative mechanisms of change (i.e., intrapersonal and interpersonal factors), and high-risk behaviors. The PI will work with an accomplished, multidisciplinary mentorship team (Drs. Spirito, Barnett, Becker, and Clark) to master four relevant areas of training: 1) effective MJ and SU interventions for CINI youth; 2) interpersonal mechanisms of adolescent SU; 3) development of technology- assisted behavioral interventions; and 4) qualitative methods for treatment development. The research and training activities outlined in this K23 award contribute to both an important public health concern and the development of a productive and independent research career for the PI. Completion of this study has the potential to advance the field by demonstrating the feasibility, acceptability, and preliminary efficacy of an adjunct technology-assisted intervention to improve outcomes among high-risk, MJ-using CINI youth.

PUBLIC HEALTH RELEVANCE: Teenagers who use marijuana and go to juvenile court are more likely to face arrest or addiction in the future. Treating their marijuana use within the court system is ideal, but treatments would need to be inexpensive and easy to deliver. This study will test a new smartphone app to help teenagers in the court system cut down on their marijuana use.

CRITIQUE 1

Candidate: 1
Career Development Plan/Career Goals & Objectives: 1
Research Plan: 2
Mentor(s), Co-Mentor(s), Consultant(s), Collaborator(s): 1
Environment and Institutional Commitment to the Candidate: 1

Overall Impact: This is a highly responsive resubmission from Dr. Sarah Helseth, an excellent candidate for a K23 award to gain skills in designing and testing substance use interventions in real world settings for youth at risk for substance use and other problems. Consistent with the first submission, Dr. Helseth presents a comprehensive and detailed training plan with mentors that she has previously collaborated with on research studies and manuscripts. Currently she is a T32 post doc fellow at the Brown University Center for Alcohol and Addictions and has several first author papers in print or under review. Institutional commitment is very high as letters from her mentors and the University attest; she will be offered an Assistant Professor position upon completion of her T32. Dr. Helseth describes an innovative research study that draws from theory and her previous experience and research and is consonant with the research of her mentors. Her research plan is to develop and assess preliminary efficacy of a theoretically-grounded, technology-based intervention for non-detained youth in the juvenile justice system in a small RCT. The app is innovative as it will be an adjunct to a one-session motivational enhancement therapy, the standard treatment in the RI juvenile justice court, and will involve intrapersonal behavior change strategies and adult-moderated networking with peers to leverage interpersonal mechanisms of behavior change mirroring the social network aspect of youths' smartphone behavior. The study uses a clear model for developing the app which involves formative work including conducting focus groups and consensus testing among youth in the target population, parents, and app developers, another innovative aspect of the proposal. The revised proposal is highly responsive to the previous review adding rigor to several aspects of the design including lengthening the assessment period, changing the recruitment plan to ensure a sufficient number of youth, dropping mediation analyses, and assessing motivation for change which may be low in this group of youth involved in the juvenile justice system. While there is no description of how the online peer forum will be moderated, limiting an understanding of dissemination potential if the intervention is found to be efficacious, the overall strength and innovativeness of the proposal, combined with the expertise of the extremely supportive mentors, moderates this weakness.

1. Candidate:

Strengths

- Dr. Helseth has received research and clinical training and is currently in her second year as a T32 post doc fellow at the Brown University Ctr for Alcohol and Addictions Studies where she has been working with several of the mentors who will continue to mentor her during the proposed K23 period.
- Dr. Helseth has 12 (5 first author) peer reviewed articles and 5 (2 as first author) under review.

Weaknesses

- None noted

2. Career Development Plan/Career Goals & Objectives:

Strengths

- Dr. Helseth's goals are clear and well-thought out and are an excellent match with the proposed mentors and the environment at the Brown School of Public Health. She proposes a main training goal to develop expertise in effective substance use treatments for high risk youth and three other goals to serve this main goal which include gaining knowledge of the interpersonal mechanisms of adolescent substance use, developing the capacity to develop and test technology-based interventions, and acquire training in qualitative methods for treatment development.
- Specific activities for each goal are detailed over the course of the five-year training period and are appropriate for meeting each goal.
- Goals include plans for development of an R01 and R21 application in the final year of the training period with primary and secondary mentors as co-investigators.

Weaknesses

- None noted.

3. Research Plan:

Strengths

- Innovative study to develop an app as an adjunct to a one session motivational enhancement therapy (the standard treatment in the RI juvenile justice court). The intervention is grounded in theories of adolescent development, social learning, and social norms and incorporates adult-moderated interpersonal peer networks to support behavior change.
- The application clearly describes the empirical and real-world smartphone behavior of youth underlying the proposed technology-based intervention for marijuana use reduction including the efficacy of accurate peer norms of substance, peer reinforcement for prosocial behavior, and gamification for engagement and achievement of substance use goals.
- In response to the previous review Dr. Helseth has revised the timeline to provide more time for development and clarifies that mentors have successfully previously worked with the contract app developer. The application carefully details the use of the Behavior Intervention Theory model to guide development and testing of the app.
- Responses to prior review are carefully considered and include the addition of a 3rd assessment at 6 months post baseline; changes to the recruitment plan to ensure a sufficient number of youth participating in the intervention at any given time; assessing motivation for decreasing marijuana use at baseline and including motivation in the moderator analysis.
- The current data analysis plan is to establish effect sizes and test for associations among potential moderators (mediation analyses are dropped in this version due to small sample size).

Weaknesses

- There is no description of how the online forum will be moderated (who, when, frequency, duration).

4. Mentor(s), Co Mentor(s), Consultant(s), Collaborator(s):

Strengths

- Dr. Anthony Spirito, the primary mentor, is a leader in the field, has an excellent track record of mentoring, and has conducted studies in the RI juvenile court where the proposed research will take place. Dr. Spirito's letter was extremely enthusiastic about mentoring Dr. Helseth.
- Drs. Barnett, Becker, and Clark co-mentors are all at Brown and are senior researchers in the field of substance use and behavioral technologies and have excellent mentoring records.
- Each of the mentors express detailed enthusiasm about Dr. Helseth's capacity to establish an independent career as a researcher examing substance use among youth.
- Dr. Helseth has collaborated with several of these mentors on research projects and manuscripts.

Weaknesses

- None noted.

5. Environment and Institutional Commitment to the Candidate:

Strengths

- Commitment to Dr. Helseth is high, she will be offered an Assistant Professorship at the Brown School of Public Health upon completion of her T32 post-doctoral fellowship which includes released time from teaching and other activities to focus on her research.
- The Brown School of Public Health and the Center for Alcohol and Addictions provides an excellent environment.

Weaknesses

- None noted.

Study Timeline:

Strengths

- Timeline has been adjusted from the previous review and appears to be appropriate to the work proposed.

Weaknesses

- None noted.

Protections for Human Subjects:

Acceptable Risks and Adequate Protections

- Human subject concerns are well described.

Data and Safety Monitoring Plan (Applicable for Clinical Trials Only):

Acceptable

- A DSMB will be convened.

Inclusion Plans:

- Sex/Gender: Distribution justified scientifically
- Race/Ethnicity: Distribution justified scientifically
- For NIH-Defined Phase III trials, Plans for valid design and analysis:
- Inclusion/Exclusion Based on Age: Distribution justified scientifically

- Includes youth ages 14 to 18 with appropriate distribution of racial and ethnic minorities. Children under 14 and adults over 18 are excluded and this is scientifically justified. Female youth will be oversampled to account for the large number of males in the RI juvenile justice court.

Vertebrate Animals:

Not Applicable (No Vertebrate Animals)

Biohazards:

Not Applicable (No Biohazards)

Resubmission:

- Highly responsive to previous reviews.

Training in the Responsible Conduct of Research:

Acceptable

Comments on Format (Required):

- Combines online and in person courses and readings.

Comments on Subject Matter (Required):

- Subject matter is appropriate for training.

Comments on Faculty Participation (Required; not applicable for mid- and senior-career awards):

- Meetings with mentors and university wide faculty through a research ethics lecture series.

Comments on Duration (Required):

- Training will continue for the duration of the award.

Comments on Frequency (Required):

- Training will occur monthly and on an as needed basis.

Budget and Period of Support:

Recommend as Requested

CRITIQUE 2

Candidate: 2
Career Development Plan/Career Goals & Objectives: 2
Research Plan: 2
Mentor(s), Co-Mentor(s), Consultant(s), Collaborator(s): 1
Environment and Institutional Commitment to the Candidate: 1

Overall Impact: This re-submitted K23 application includes many strengths including a strong candidate in Dr. Helseth, excellent training plan and significant and innovative research component. Dr. Helseth has an excellent training background. She has been productive with 12 peer reviewed publications (5 first author), with several more under review. The mentorship team, which includes Drs. Spirito, Barnett, Becker and Clark, is excellent. Dr. Helseth's career development plan has four clearly

articulated training objectives: 1) develop expertise in effective cannabis and substance use treatments with court-involved youth; 2) extend her knowledge of interpersonal mechanisms of youth substance use; 3) gain skills in technology-based interventions; and 4) acquire specialized training in qualitative methods. The overall aim of Dr. Helseth's proposed research is to develop and pilot test an adjunct mHealth app designed to promote cannabis use reduction among court-involved, non-incarcerated youth. The study will be conducted in 3 phases: intervention development through stakeholder interviews; beta testing the intervention; and pilot testing to establish initial feasibility, acceptability and efficacy relative to TAU). The methodological rigor of the study is quite strong, including random assignment, intent-to-treat design and several other aspects. This is a very strong proposal.

1. Candidate:

Strengths

- Since her original submission, the candidate has continued to demonstrate very good productivity with 12 peer-reviewed articles (5 as lead author), with 5 more under review (2 as lead). She also notes that 6 of these papers and 6 national presentations address substance use samples and treatments.
- Prior research and clinical training experiences, including dissertation at FIU and post-doctoral fellowship at Brown were of high quality.
- Dr. Helseth appears committed to meet program objectives to become an independent scientist.
- Very enthusiastic letters of support that support candidate's potential to develop as an independent researcher.

Weaknesses

- None noted.

2. Career Development Plan/Career Goals & Objectives:

Strengths

- Dr. Helseth's objectives for training/career goals were clearly articulated, and sound rationale provided for each. The four objectives included obtaining expertise in cannabis treatments for high risk CINI youth, building on knowledge of interpersonal mechanisms of substance use, gaining skills in technology assisted interventions, and acquire qualitative skills training.
- Content, scope, phasing and duration of training seem appropriate.

Weaknesses

- None noted.

3. Research Plan:

Strengths

- The research plan describes the need for adjunct interventions, and identifies several gaps in the literature that provide a basis for the proposed study including: 1) high rates of cannabis use in CINI youth; 2) identification of barriers to youth SU treatment; 3) selection of treatment approach, with evidence favoring motivational enhancement; and 4) providing a rationale for technology-based interventions, particularly for cannabis use.
- The intervention is based on social learning theory, and the intervention leverages peer influences to reduce cannabis use.

- This study is innovative in several ways. It is among the first to address cannabis use in court involved youth. It is one of the first to use an MET approach within this context. It will be the first study to build a user-driven smartphone app to reduce cannabis use. This study will also be the first to leverage interpersonal mechanisms to change cannabis use in court involved youth. Only two prior studies have explored the feasibility and efficacy of mobile apps as adjuncts for substance use in youth.
- Rhode Island family court supports the project.
- Methodological rigor is supported by several elements including use of random assignment, intent-to-treat design, over-sampling of female youth, use of urine drug screens to validate self-reported substance use, and rigorous intervention development and field testing.
- Responsiveness to the previous reviews has improved the methodological rigor through improved pilot data and longer follow-up period.

Weaknesses

- Some good strategies (e.g., enrolling after 4 families are available) are introduced to ensure enough youth are involved at one time, but how will it be handled if the critical number of participants can't be met for a sustained period, or if several youth drop out leaving only one or two?
- Although the investigator has a fairly good rationale for including MET based TAU, there is still the potential to find no (or minimal) additive effects of the app given similarities with TAU. Granted, it is understood that this is a pilot trial and not meant to be fully powered, thus this concern may be minimal.
- It is noted that ANCOVA will be used to test outcomes, but it is not clear how this will be done using urine drug screen data.

4. Mentor(s), Co Mentor(s), Consultant(s), Collaborator(s):

Strengths

- Dr. Spirito (Primary mentor) has a long and extensive history of mentoring scholars at many levels.
- Each of the mentors has a history of training post-doctoral fellows, and each is quite accomplished in areas directly relevant to Dr. Helseth's proposed training plan.
- Training aims/goals for all mentors (Spirito, Barnett, Becker, Clark) are thoroughly detailed.
- Excellent support letters are provided.

Weaknesses

- None noted.

5. Environment and Institutional Commitment to the Candidate:

Strengths

- The environment at Brown and commitment to the candidate are excellent.
- As all of the mentors are located at Brown, Dr. Helseth should have ample physical access to them.
- Per letter from Dr. Monti, Dr. Helseth will be provided with protected time to engage in the activities of the K award. She will be awarded a faculty appointment at Brown University School of Health (Assistant Professor). She can use 100% of her time for research.

- Center for Addiction and Alcohol Studies has a long history of T-32 and other training programs.

Weaknesses

- None noted.

Study Timeline:

Strengths

- The timeline is very thorough.

Weaknesses

- None noted.

Protections for Human Subjects:

Acceptable Risks and Adequate Protections

- Risks and appropriate protections are well described.

Data and Safety Monitoring Plan (Applicable for Clinical Trials Only):

Acceptable

- o A DSMB will be convened.

Inclusion Plans:

- Sex/Gender: Distribution justified scientifically
- Race/Ethnicity: Distribution not justified scientifically
- For NIH-Defined Phase III trials, Plans for valid design and analysis: Not applicable
- Inclusion/Exclusion Based on Age: Distribution justified scientifically
- Youth age 13-17 make up the CINI population. It is expected that more males will make up the population, so female participants will be over-sampled. The racial/ethnic makeup will match the CINI population in Rhode Island.

Vertebrate Animals:

Not Applicable (No Vertebrate Animals)

Biohazards:

Not Applicable (No Biohazards)

Resubmission:

- Dr. Helseth's application was quite responsive to prior reviews. Notably, she increased the number of participants in her pilot work and included a 6-month follow-up in her planned research.

Training in the Responsible Conduct of Research:

Acceptable

Comments on Format (Required):

- Citi, NIH online Protecting Human Research Participants, Reproducibility Training and Good Clinical Practice will be completed online. Yearly Brown University EIPR seminar series, Brown Ethics and Responsible Conduct of Research course, will be conducted in person. An additional reading list is also provided.

Comments on Subject Matter (Required):

- Conflicts of interest, mentor/mentee roles/relationships, scientist as a responsible member of society, and fiscal responsibility in sponsored research are covered in the BEACORE course.

Comments on Faculty Participation (Required; not applicable for mid- and senior-career awards):

- Primary mentor Spirito will oversee RCR training with updates biannually to the full mentorship team. BEACORE seminars are faculty led.

Comments on Duration (Required):

- The BEACORE course is 8 hours yearly.

Comments on Frequency (Required):

- NIH Annual Review of Ethics Cases and CITI re-training will be completed yearly.

Budget and Period of Support:

Recommend as Requested

CRITIQUE 3

Candidate: 1
Career Development Plan/Career Goals & Objectives: 2
Research Plan: 1
Mentor(s), Co-Mentor(s), Consultant(s), Collaborator(s): 1
Environment and Institutional Commitment to the Candidate: 1

Overall Impact: In this resubmitted application, Dr. Helseth successfully addressed the concerns of her original submission by publishing data in the areas that will serve as the basis for this mentorship grant, by collecting additional preliminary data, and by clarifying other concerns that were noted in the original review. Dr. Helseth continues to be an exceptionally strong candidate for the K23 award, having relevant past experience with youth and interpersonal / intrapersonal factors related to risk. Dr. Helseth has published 12 manuscripts and has several others in various states of publication. The long-term career goals are appropriate and the additional training that Dr. Helseth will receive during the K-award period will fill important gaps in her skill set that should adequately prepare her to become an independent scientist. The research plan is based on scientifically rigorous research and proposes to develop a mobile intervention that can be used to extend existing treatments for high-risk youth (CINI) who use marijuana (MJ). The primary and co-mentors are exceptionally well-suited to provide Dr. Helseth with the training she will need, although the number of meetings that are scheduled may interfere with her productivity, so finding a way to streamline those meetings may be helpful. The mentors and institution have a strong record of successful mentorship with the K-award mechanism.

1. Candidate:

Strengths

- Prior experience with at-risk children and peer influences on behavior.

- Received a fellowship and internal award to fund dissertation research developing a peer-facilitated intervention implemented in a community-based setting targeting low-income, minority youth.
- Completed an internship at MUSC further exploring the sustainability of evidence-based interventions in a community setting.
- Went on as a post-doctoral fellow at Brown University under T32 fellowship to study adolescent substance use (SU), where first exposed to mHealth interventions.
- Submitted 4 manuscripts (1 as first author) with proposed mentors to gain additional relevant experience related to the proposed research trajectory.
- Began conducting clinical work with the target population (MJ /SU CINI youth).
- Appropriately identifies skill deficits that will be acquired during this mentored award, and will help the applicant become an independent researcher.

Weaknesses

- Some discrepancy about the number of new manuscripts and presentations related to the proposed research (4 in some places, 6 in others, with regard to manuscripts).

2. Career Development Plan/Career Goals & Objectives:

Strengths

- Appropriate overall career goal of working in a school of public health or academic research setting, focusing on a SU interventions for youth in risky settings.
- Short term objectives outlined in this section should prepare Dr. Helseth to become an independent researcher, and thereby achieve her overall goal.
- The mentorship team is exceptional, and each serve unique roles for satisfying the skills that Dr. Helseth seeks to develop during this mentored award.
- Plans for presenting and publishing research are appropriate (2 first authored of each per year).
- Semi-annual progress report given to mentors for feedback on progress with K23 activities.

Weaknesses

- There are 15 planned meetings per month (not including the semi-annual meeting with the entire mentorship team) that will take place throughout the entire award period. Some concerns about this taking away valuable time from engaging in the other proposed research and training activities.

3. Research Plan:

Strengths

- Focus on interpersonal and intrapersonal factors in court-involved not incarcerated (CINI) MJ youth is well supported by previous literature.
- Scientific rigor of previous research on MET for MJ in teens is strong and given limitations to other more time-intensive evidence-based interventions, MET is well justified as the primary intervention that will be supplemented with technology.
- Scientifically rigorous research leads to a strong rationale for focusing on technology delivered interventions involving peers for adolescent SU.
- The project is innovative because of its focus on CINI youth, creating a technology-based adjunct that focuses on peer support, to be used in conjunction with brief MET.

- Scientifically rigorous research design including feedback from key stakeholders during early stage development, and ending with a small RCT, with an appropriate TAU control condition, to test preliminary efficacy.
- Compelling preliminary research suggesting the potential feasibility and acceptability of the proposed approach.
- Analysis plan is appropriate for each stage of proposed research.

Weaknesses

- Nothing of substance noted.

4. Mentor(s), Co Mentor(s), Consultant(s), Collaborator(s):

Strengths

- Primary Mentor (Spirito) has a successful track record of training post-doctoral fellows to become independent researchers and has the expertise of MJ treatment with CINI youth, particularly using MET which is proposed for this project. Dr. Spirito has demonstrated his commitment to training Dr. Helseth by including her on manuscripts and conference presentations recently.
- Co-Mentor (Barnett) has prior experience mentoring K-awardees and has expertise in adolescent SU and interpersonal processes related to use. Dr. Barnett also has some experience with mHealth interventions as well.
- Co-Mentor (Becker) has served as a mentor for Dr. Helseth previously but for this project would provide more guidance on the development of mHealth interventions. Dr. Becker has extensive experience with technology development and is the PI on several grants, including 2 center grants, focused on technology transfer and training in community settings for SU treatment. Dr. Becker was also a recipient of a K23, mentored by Dr. Spirito, that ended in 2017.
- Co-Mentor (Clark) has expertise in qualitative design methods and has experience mentoring researchers in these designs.

Weaknesses

- None noted.

5. Environment and Institutional Commitment to the Candidate:

Strengths

- Brown University, and the Center for Alcohol and Addiction Studies in particular, are exceptional environments for completing the proposed activities. Many K-awardees have received training in this strong environment.
- The Rhode Island Family Court will be the recruitment site. The primary mentor has an ongoing relationship with this site and the letter of support leads to confidence in the project being successfully completed here.

Weaknesses

- None noted.

Study Timeline:

Strengths

- There seems to be adequate time allocated to the various aims and activities of the proposal, including app development time.
- The recruitment site, RIFC, has an adequate number of potentially eligible youth leading to confidence in the projected recruitment timeline.
- The timeline for other training activities is realistic.

Weaknesses

- None noted.

Protections for Human Subjects:

Acceptable Risks and Adequate Protections

- Appropriate protections are described.

Data and Safety Monitoring Plan (Applicable for Clinical Trials Only):

Acceptable

- o The DSMP is appropriate, and a DSMB will be convened for the Phase 3 RCT.

Inclusion Plans:

- Sex/Gender: Distribution justified scientifically
- Race/Ethnicity: Distribution justified scientifically
- For NIH-Defined Phase III trials, Plans for valid design and analysis: Not applicable
- Inclusion/Exclusion Based on Age: Distribution justified scientifically
- Girls will be oversampled in the early phases to ensure representation, but the majority of CINI youth are male. Racial and ethnic targets are justified, and recruiting children is the purpose of the proposed research.

Vertebrate Animals:

Not Applicable (No Vertebrate Animals)

Biohazards:

Not Applicable (No Biohazards)

Resubmission:

- Has more experience in the proposed area than with the first submission, working with MJ users who are court-involved, presented research at national conferences, and collaborated on manuscripts with mentors.
- Updated coursework to better align with the goals of the proposed research.
- Added time and prior experience to alleviate concerns about the TECH development timeline, added methods to prioritize features and ensure that there is sufficient peer presence.
- Added longer 6-month follow up and increased samples size to 20.

Training in the Responsible Conduct of Research:

Acceptable

Comments on Format (Required):

- In person and online formats.

Comments on Subject Matter (Required):

- Appropriate topics, broadly speaking, will be addressed (e.g., conflicts of interest, scientific misconduct, fiscal responsibility) but also topics more relevant to the population and topics that Dr. Helseth is studying (e.g., working with substance users in the criminal justice system, adolescents, etc.).

Comments on Faculty Participation (Required; not applicable for mid- and senior-career awards):

- Faculty will teach the responsible conduct of research seminars that are available at the institution.
- Dr. Helseth will meet monthly to discuss RCR issues with her primary mentor/faculty member as well.

Comments on Duration (Required):

- Two of the seminars involve 8, 1-hour sessions that will be repeated throughout the K-award period.

Comments on Frequency (Required):

- The seminars will be completed weekly for 8 weeks each (two different 8 week seminars were described).
- The online trainings will occur annually.

Budget and Period of Support:

Recommend as Requested

- No overlaps were identified.

THE FOLLOWING SECTIONS WERE PREPARED BY THE SCIENTIFIC REVIEW OFFICER TO SUMMARIZE THE OUTCOME OF DISCUSSIONS OF THE REVIEW COMMITTEE, OR REVIEWERS' WRITTEN CRITIQUES, ON THE FOLLOWING ISSUES:

PROTECTION OF HUMAN SUBJECTS: ACCEPTABLE

INCLUSION OF WOMEN PLAN: ACCEPTABLE

INCLUSION OF MINORITIES PLAN: ACCEPTABLE

INCLUSION ACROSS THE LIFESPAN PLAN: ACCEPTABLE

COMMITTEE BUDGET RECOMMENDATIONS: The budget was recommended as requested.

Footnotes for 1 K23 DA048062-01A1; PI Name: Helseth, Sarah Ashley

+ Derived from the range of percentile values calculated for the study section that reviewed this application.

NIH has modified its policy regarding the receipt of resubmissions (amended applications). See Guide Notice NOT-OD-14-074 at http://grants.nih.gov/grants/guide/notice-files/NOT-OD-14-074.html. The impact/priority score is calculated after discussion of an application by averaging the overall scores (1-9) given by all voting reviewers on the committee and multiplying by 10. The criterion scores are submitted prior to the meeting by the individual reviewers assigned to an application, and are not discussed specifically at the review meeting or calculated into the overall impact score. Some applications also receive a percentile ranking. For details on the review process, see http://grants.nih.gov/grants/peer_review_process.htm#scoring.

MEETING ROSTER

Interventions to Prevent and Treat Addictions Study Section
Risk, Prevention and Health Behavior Integrated Review Group
CENTER FOR SCIENTIFIC REVIEW
IPTA
06/06/2019 - 06/07/2019

Notice of NIH Policy to All Applicants: Meeting rosters are provided for information purposes only. Applicant investigators and institutional officials must not communicate directly with study section members about an application before or after the review. Failure to observe this policy will create a serious breach of integrity in the peer review process, and may lead to actions outlined in NOT-OD-14-073 at https://grants.nih.gov/grants/guide/notice-files/NOT-OD-14-073.html and NOT-OD-15-106 at https://grants.nih.gov/grants/guide/notice-files/NOT-OD-15-106.html, including removal of the application from immediate review.

CHAIRPERSON(S)

WALTON, MAUREEN A, PHD, MPH
PROFESSOR
DEPARTMENT OF PSYCHIATRY
UNIVERSITY OF MICHIGAN
ANN ARBOR, MI 48109

MEMBERS

BRADIZZA, CLARA M, PHD
PROFESSOR
SCHOOL OF SOCIAL WORK
UNIVERSITY AT BUFFALO
STATE UNIVERSITY OF NEW YORK
BUFFALO, NY 14203

CALHOUN, PATRICK S, PHD *
PROFESSOR
DEPARTMENT OF PSYCHIATRY AND BEHAVIORAL SCIENCES
SCHOOL OF MEDICINE
DUKE UNIVERSITY
DURHAM, NC 27705

FRIEDMANN, PETER D, MD, MPH
PROFESSOR
DEPARTMENT OF MEDICINE
UNIVERSITY OF MASSACHUSETTS
MEDICAL SCHOOL - BAYSTATE
SPRINGFIELD, MA 01107

GARRISON, KATHLEEN A., PHD *
ASSISTANT PROFESSOR
DEPARTMENT OF PSYCHIATRY
YALE SCHOOL OF MEDICINE
NEW HAVEN , CT 06510

GRAY, KEVIN M, MD
PROFESSOR
DEPARTMENT OF PSYCHIATRY
AND BEHAVIORAL SCIENCES
MEDICAL UNIVERSITY OF SOUTH CAROLINA
CHARLESTON, SC 29425

HARTZLER, BRYAN J, PHD *
RESEARCH SCIENTIST
ALCOHOL AND DRUG ABUSE INSTITUE
UNIVERSITY OF WASHINGTON
SEATTLE, WA 98105

HEIL, SARAH H, PHD
PROFESSOR
DEPARTMENT OF PSYCHIATRY
COLLEGE OF MEDICINE
UNIVERSITY OF VERMONT
BURLINGTON, VT 05401

HITSMAN, BRIAN L, PHD
ASSOCIATE PROFESSOR
DEPARTMENT OF PREVENTIVE MEDICINE
FEINBERG SCHOOL OF MEDICINE
NORTHWESTERN UNIVERSITY
CHICAGO, IL 60611

JACOBSON, KAREN R, MD, MPH *
ASSISTANT PROFESSOR
DEPARTMENT OF MEDICINE
BOSTON UNIVERSITY SCHOOL OF MEDICINE
BOSTON, MA 02118

LARSON, MARY JO, PHD
SENIOR SCIENTIST
INSTITUTE FOR BEHAVIORAL HEALTH
HELLER SCHOOL FOR SOCIAL POLICY AND MANAGEMENT
BRANDEIS UNIVERSITY
WALTHAM, MA 02454

LEDGERWOOD, DAVID M, PHD *
ASSOCIATE PROFESSOR
DEPARTMENT OF PSYCHIATRY AND
BEHAVIORAL NEUROSCIENCES
SCHOOL OF MEDICINE
WAYNE STATE UNIVERSITY
DETROIT, MI 48201

LEONARD, NOELLE R, PHD *
SENIOR RESEARCH SCIENTIST
SILVER SCHOOL OF SOCIAL WORK
NEW YORK UNIVERSITY
NEW YORK, NY 10003

MCGOVERN, MARK P, PHD
PROFESSOR
DEPARTMENT OF PSYCHIATRY
AND BEHAVIORAL SCIENCES
STANFORD UNIVERSITY
PALO ALTO, CA 93404

MENDELSON, TAMAR, PHD
ASSOCIATE PROFESSOR
DEPARTMENT OF MENTAL HEALTH
JOHNS HOPKINS BLOOMBERG SCHOOL OF PUBLIC HEALTH
BALTIMORE, MD 21205

NAHVI, SHADI, MD *
ASSOCIATE PROFESSOR
DEPARTMENT OF MEDICINE AND PSYCHIATRY
ALBERT EINSTEIN COLLEGE OF MEDICINE
MONTEFIORE MEDICAL CENTER
BRONX, NY 10467

OLIVETO, ALISON, PHD
PROFESSOR AND VICE CHAIR FOR RESEARCH
DEPARTMENT OF PSYCHIATRY
UNIVERSITY OF ARKANSAS FOR MEDICAL SCIENCES
LITTLE ROCK, AR 72205

ONCKEN, CHERYL A, MD, MPH
PROFESSOR
DEPARTMENT OF MEDICINE AND
OBSTETRICS/GYNECOLOGY
UNIVERSITY OF CONNECTICUT HEALTH CENTER
FARMINGTON, CT 06030

OSILLA, KAREN C, PHD
SENIOR BEHAVIORAL SCIENTIST
RAND CORPORATION
SANTA MONICA, CA 90404

RAIFF, BETHANY R, PHD *
ASSOCIATE PROFESSOR
DEPARTMENT OF PSYCHOLOGY
ROWAN UNIVERSITY
GLASSBORO, NJ 08028

SHEFFER, CHRISTINE ELIZABETH, PHD *
ASSOCIATE PROFESSOR
DEPARTMENT OF HEALTH BEHAVIOR
ROSWELL PARK COMPREHENSIVE CANCER CENTER
BUFFALO, NY 14203

STEIN, MICHAEL D, MD
PROFESSOR AND CHAIR
DEPARTMENT OF HEALTH LAW, POLICY AND MANAGEMENT
BOSTON UNIVERSITY SCHOOL OF PUBLIC HEALTH
BOSTON, MA 02118

STOOPS, WILLIAM W, PHD
PROFESSOR
DEPARTMENT OF BEHAVIORAL SCIENCE
UNIVERSITY OF KENTUCKY
LEXINGTON, KY 40536

TINDLE, HILARY A, MD, MPH
ASSOCIATE PROFESSOR
DIVISION OF INTERNAL MEDICINE AND PUBLIC HEALTH
VANDERBILT UNIVERSITY MEDICAL CENTER
NASHVILLE, TN 37203

TSOH, JANICE Y, PHD
PROFESSOR
DEPARTMENT OF PSYCHIATRY
LANGLEY PORTER PSYCHIATRIC INSTITUTE
UNIVERSITY OF CALIFORNIA SAN FRANCISCO
SAN FRANCISCO, CA 94143

VELASQUEZ, MARY M, PHD
CENTENNIAL PROFESSOR AND DIRECTOR
HEALTH BEHAVIOR RESEARCH
AND TRAINING INSTITUTE
SCHOOL OF SOCIAL WORK
UNIVERSITY OF TEXAS AT AUSTIN
AUSTIN, TX 78712

YURGELUN-TODD, DEBORAH A, PHD *
PROFESSOR
DEPARTMENT OF PSYCHIATRY
SCHOOL OF MEDICINE
UNIVERSITY OF UTAH
SALT LAKE CITY, UT 84108

SCIENTIFIC REVIEW OFFICER

MINTZER, MIRIAM, PHD
SCIENTIFIC REVIEW OFFICER
CENTER FOR SCIENTIFIC REVIEW
NATIONAL INSTITUTES OF HEALTH
BETHESDA, MD 20892

EXTRAMURAL SUPPORT ASSISTANT

FAYEMIWO, TOLU
EXTRAMURAL SUPPORT ASSISTANT
CENTER FOR SCIENTIFIC REVIIEW
NATIONAL INSTITUTES OF HEALTH
BETHESDA, MD 20892

* Temporary Member. For grant applications, temporary members may participate in the entire meeting or may review only selected applications as needed.

Consultants are required to absent themselves from the room during the review of any application if their presence would constitute or appear to constitute a conflict of interest.
